# Effect of the identification group size and image resolution on the diagnostic performance of metabolic Alzheimer’s disease-related pattern

**DOI:** 10.1186/s13550-023-01001-5

**Published:** 2023-05-24

**Authors:** Eva Štokelj, Petra Tomše, Tadej Tomanič, Vijay Dhawan, David Eidelberg, Maja Trošt, Urban Simončič, for the Alzheimer’s Disease Neuroimaging Initiative

**Affiliations:** 1 Faculty of Mathematics and Physics, University of Ljubljana, Jadranska ulica 19, 1000 Ljubljana, Slovenia; 2 Department of Nuclear Medicine, University Medical Centre Ljubljana, Zaloška cesta 7, 1000 Ljubljana, Slovenia; 3 Center for Neurosciences, The Feinstein Institute for Medical Research, 350 Community Dr, Manhasset, NY 11030 USA; 4 Department of Neurology, University Medical Centre Ljubljana, Zaloška cesta 2, 1000 Ljubljana, Slovenia; 5 Faculty of Medicine, University of Ljubljana, Vrazov trg 2, 1000 Ljubljana, Slovenia; 6 Jožef Stefan Institute, Jamova cesta 39, 1000 Ljubljana, Slovenia

**Keywords:** Alzheimer’s disease, Metabolic brain pattern, 2-[^18^F]FDG-PET, Image resolution, Cohort size, Network analysis

## Abstract

**Background:**

Alzheimer’s disease-related pattern (ADRP) is a metabolic brain biomarker of Alzheimer’s disease (AD). While ADRP is being introduced into research, the effect of the size of the identification cohort and the effect of the resolution of identification and validation images on ADRP’s performance need to be clarified.

**Methods:**

240 2-[^18^F]fluoro-2-deoxy-d-glucose positron emission tomography images [120 AD/120 cognitive normals (CN)] were selected from the Alzheimer's disease neuroimaging initiative database. A total of 200 images (100 AD/100 CN) were used to identify different versions of ADRP using a scaled subprofile model/principal component analysis. For this purpose, five identification groups were randomly selected 25 times. The identification groups differed in the number of images (20 AD/20 CN, 30 AD/30 CN, 40 AD/40 CN, 60 AD/60 CN, and 80 AD/80 CN) and image resolutions (6, 8, 10, 12, 15 and 20 mm). A total of 750 ADRPs were identified and validated through the area under the curve (AUC) values on the remaining 20 AD/20 CN with six different image resolutions.

**Results:**

ADRP’s performance for the differentiation between AD patients and CN demonstrated only a marginal average AUC increase, when the number of subjects in the identification group increases (AUC increase for about 0.03 from 20 AD/20 CN to 80 AD/80 CN). However, the average of the lowest five AUC values increased with the increasing number of participants (AUC increase for about 0.07 from 20 AD/20 CN to 30 AD/30 CN and for an additional 0.02 from 30 AD/30 CN to 40 AD/40 CN). The resolution of the identification images affects ADRP’s diagnostic performance only marginally in the range from 8 to 15 mm. ADRP’s performance stayed optimal even when applied to validation images of resolution differing from the identification images.

**Conclusions:**

While small (20 AD/20 CN images) identification cohorts may be adequate in a favorable selection of cases, larger cohorts (at least 30 AD/30 CN images) shall be preferred to overcome possible/random biological differences and improve ADRP’s diagnostic performance. ADRP’s performance stays stable even when applied to the validation images with a resolution different than the resolution of the identification ones.

## Background

Alzheimer’s disease (AD) is the most common neurodegenerative brain disorder, affecting mostly the older population [1, 2]. Although other neurodegenerative syndromes have different clinical courses and outcomes, the early clinical presentations may overlap. Therefore, an objective biomarker is needed to confirm the diagnosis at its early stages. Such a biomarker would improve treatment efficiency and prognostic accuracy in subjects with dementia as well as improve the accuracy of diagnosis in the research trials [3].

The so-called AD-related pattern (ADRP) is a metabolic brain biomarker of AD, which has been validated in previous work [4–8]. ADRP was derived with the application of a scaled subprofile model/principal component analysis (SSM/PCA) in groups of 2-[^18^F]fluoro-2-deoxy-d-glucose positron emission tomography (2-[^18^F]FDG-PET) brain images [9] of AD patients and cognitively normal subjects (CN).

2-[^18^F]FDG-PET imaging can detect metabolic brain changes in patients with neurodegenerative disorders earlier than structural brain imaging [10]. It measures glucose consumption in brain tissue and is a proxy for neuronal activity and an index of synaptic function and density [11]. Brain metabolism is higher in regions with more vivid synaptic activity, like the brain cortex and deep brain nuclei, and lower in regions with less activity, as well as in regions affected by neurodegeneration [12].

SSM/PCA decomposes FDG distribution in the brain, shown in 2-[^18^F]FDG-PET images, into principal components (PCs), which present normal and abnormal uptake. PCs represent brain regions that are functionally related and allow the detection of specific disease-related metabolic patterns, such as ADRP [6, 13, 14].

An unresolved impediment for the translation of ADRP and other SSM/PCA based network patterns into clinical practice is presented by the need to evaluate systematically pattern’s stability regarding the number of analyzed 2-[^18^F]FDG-PET images and variations in technical parameters of the images acquired on different scanners. Previous studies have shown that image reconstruction algorithms [15–17] or different image preprocessing software [18] have a minor impact on the neurodegenerative disease-specific metabolic patterns. However, so far, the effect of the size of the pattern identification groups nor the image resolutions on the pattern’s diagnostic performance has been thoroughly investigated.

In this study, we aimed to assess the effect of two critical parameters on ADRP diagnostic performance: (i) the size of the identification group and (ii) the resolution of the 2-[^18^F]FDG-PET images in identification and validation groups. Our findings could further be used in other SSM based analyses.

## Subjects and methods

### Subjects’ selection

We analyzed 2-[^18^F]FDG-PET brain images obtained from 240 subjects from the Alzheimer’s Disease Neuroimaging Initiative (ADNI) database (https://adni.loni.usc.edu/). The ADNI was launched in 2003 as a public–private partnership led by Principal Investigator Michael W. Weiner, MD. The primary goal of ADNI has been to test whether serial magnetic resonance imaging (MRI), PET, other biological markers, and clinical and neuropsychological assessment can be combined to measure the progression of mild cognitive impairment (MCI) and early Alzheimer’s disease (AD). Images were selected randomly from the ADNI database, fulfilling the criteria described below.

Cohort A consisted of images from 100 AD patients and 100 CN patients and was used for the identification of ADRP patterns, whereas Cohort B included 20 AD patients and 20 CN, which were used for ADRP validation.

Recording in a tomograph yielding a final image resolution of 6 mm full width at half maximum (FWHM) or less was a selection/inclusion criterion for the study. For both cohorts, only patients with AD diagnosis clinically confirmed at all follow-up sessions were selected. Similarly, CN were selected among subjects who were confirmed as cognitively normal at all follow-up sessions. Additional criteria for Cohort B were matching in age, gender, and disease duration with Cohort A.

The 2-[^18^F]FDG-PET images of AD patients were checked for severe brain atrophy, which could affect the identification of disease-related pattern, using an in-house computer program written in Matlab R2020a. Atrophy volume was calculated with the underlying assumption that voxels with intensity values below 30% of the maximal value within the brain represented cerebrospinal fluid or brain atrophy. Potential members of the patient groups were excluded if their atrophy volume exceeded by more than 10% the mean atrophy volume in the CN group, whereupon we repeated the selection from within the ADNI database. More details on our PET-based atrophy screening algorithm and its validation against the established medial temporal lobe atrophy (MTA) [19] score can be found in Appendix 2.

Demographic data of the selected subjects in identification Cohort A and validation Cohort B are presented in Table 1.

**Table 1:** Demographic characteristics of subjects selected from the ADNI database. Images from Cohort A were used for ADRP identification, whereas images from Cohort B were used for ADRP validation

	Cohort A		Cohort B	
	AD	CN	AD	CN
N	100	100	20	20
Gender (M/F)	50/50	50/50	10/10	10/10
Age (yrs)*	76.6 ± 6.7	76.6 ± 6.0	76.6 ± 5.8	76.6 ± 5.2
Disease duration (yrs)*	5.1 ± 3.0	–	5.1 ± 2.8	–
MMSE*	23.3 ± 3.0	28.7 ± 1.7	22.9 ± 3.0	29.2 ± 1.1

*Age, Disease duration and MMSE are given as mean ± standard deviation

### Image acquisition protocol

All images were collected from the ADNI database and had been thus acquired and preprocessed with the protocol requested by ADNI. In brief, 30–60 min prior to the start of scanning, all participants received intravenously administered FDG with the activity of 185 MBq. Dynamic brain scans of six 5-min frames were acquired, co-registered, and averaged to minimize the motion artifacts and then re-oriented into a standard 160 × 160 × 96 voxel image grid, having 1.5 mm cubic voxels. They were acquired on different scanners from two manufacturers (General Electric and Siemens; Table 2). CN and AD subjects were similarly distributed across different scanners. We believe that various scanners would not affect our survey, since images were harmonized with same normalization and different smoothing, based on scanner resolutions. All scans were reconstructed with OSEM iterative reconstruction algorithm using parameters specific to the system used for scanning (https://adni.loni.usc.edu/methods/pet-analysis-method/pet-analysis/).

**Table 2:** Scanner resolutions. Effective scanner resolution (ESR) and Gaussian filters are used for image smoothing to achieve different final resolutions

Scanner	ESR	FWHM of Gaussian filter used for smoothing					
		Final resolution 6 mm	Final resolution 8 mm	Final resolution 10 mm	Final resolution 12 mm	Final resolution 15 mm	Final resolution 20 mm
HRRT (Siemens)	4.5/4.5	3.3/3.4	6.0/6.0	8.7/8.7	10.9/10.9	14.1/14.2	19.4/19.4
Biograph HiRes/mCT /1093/1094/1080 (Siemens)	5.5/5.5	2.3/2.4	5.5/5.5	8.3/8.4	10.6/10.7	13.9/13.9	19.2/19.2
Discovery 600, 690, RX, STE (General Electric)	5.5/6.0	2.3/0.7	5.5/5.0	8.3/8.0	10.6/10.4	13.9/13.8	19.2/19.1
HR + (Siemens)	6.0/6.0	0.3/0.7	5.0/5.0	8.0/8.0	10.4/10.4	13.8/13.8	19.1/19.1

All values in the table are full width at half maximum, presented for in-plane/axial and are given in mm

### Sizes of identification groups

To study the effect of the identification group size on the ADRP, subjects were randomly selected from the identification Cohort A to groups of different sizes, following the rule that an average age and average disease duration should remain unchanged, 76.6 years, 5.1 years, respectively, and that gender was balanced in all groups. The following 125 groups with five different sizes were created:

- 25 groups with 20 AD/20 CN subjects,
- 25 groups with 30 AD/30 CN subjects,
- 25 groups with 40 AD/40 CN subjects,
- 25 groups with 60 AD/60 CN subjects and
- 25 groups with 80 AD/80 CN subjects.

Twenty-five replications of each of the five different group sizes were used to reduce the effect of biological variability on the final result. Description of algorithm for assigning subjects to different identification groups and demographic data for all groups is presented in Appendix 1.

Validation Cohort B did not undergo the grouping process described for Cohort A but comprised of 20 AD patients and 20 CN, as originally selected from the database.

### Image resolutions

To evaluate the effect of image resolution on ADRP performance, we preprocessed the 125 groups with identification images (Cohort A) and the validation image group (Cohort B) to obtain fictionally made six different image resolutions: 6, 8, 10, 12, 15, and 20 mm FWHM. 6 mm is the best achievable resolution in our scanner selection and 20 mm is above the upper limit of the image resolutions in previously identified ADRP patterns [4–7, 20]. For this purpose, we spatially normalized each image with a standard Montreal Neurological Institute (MNI)-based PET template and smoothed it with a Gaussian filter of a particular width, depending on the original scanner resolution. FWHMs of filters used for smoothing were calculated as a square root of the squared desired final resolution minus the squared effective scanner resolution (assuming that the FWHM adds in squares). Filter FWHMs for smoothing the images from each selected scanner are presented in Table 2. Based on six final image resolutions, six subgroups were formed from each of the 125 previously created groups of identification subjects (750 subgroups) and the one validation group (6 subgroups). Image normalization and smoothing were done in Statistical parametric mapping 12 (SPM12; Institute of Neurology, UCL, London, UK) software running in Matlab R2020a (MathWorks Inc., Natick, MA).

**Fig. 1:**
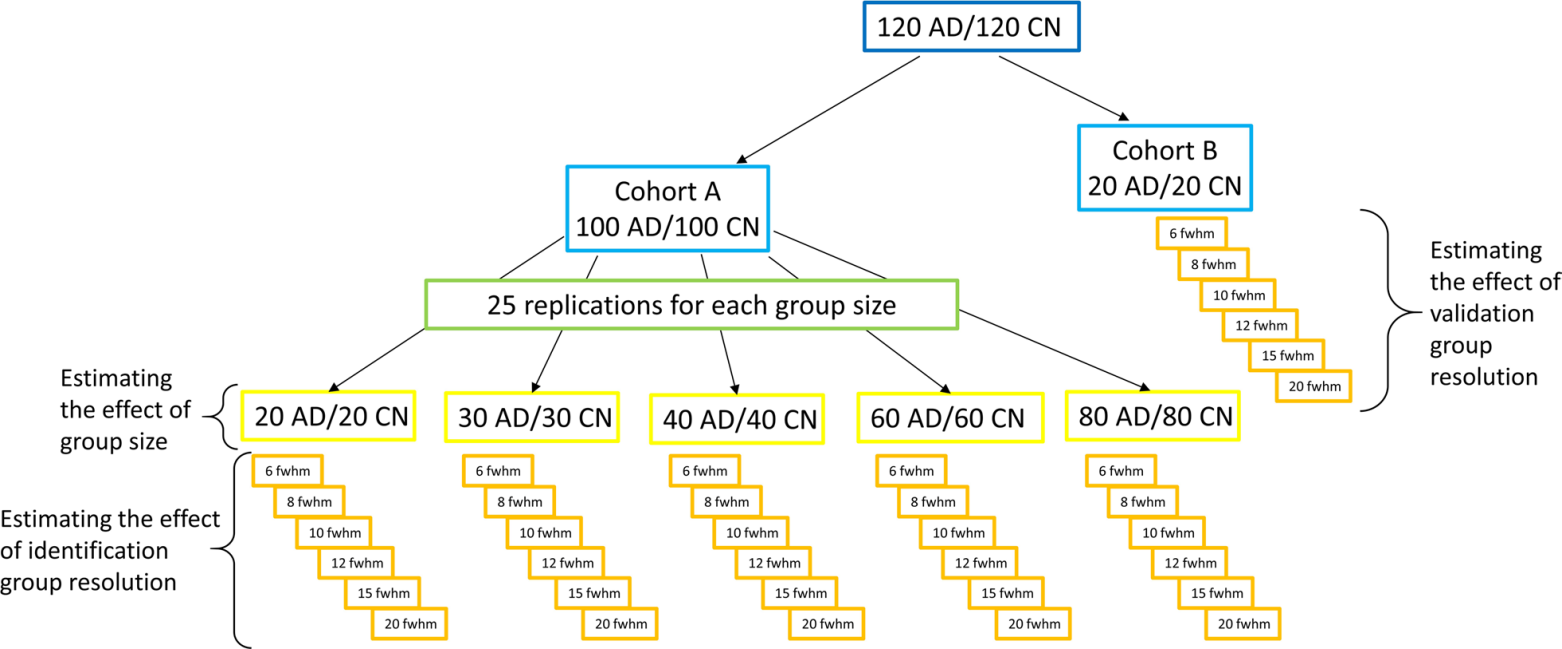
Group formation. All patients from ADNI were divided into two cohorts. From the identification Cohort A, groups of different sizes were formed, each with 25 replications. Each such group was further preprocessed to obtain 6 groups with the same subjects but different image resolutions. From validation Cohort B, 6 different image resolution groups were made

A schematic presentation of group formation and preprocessing for the identification of various versions of ADRP is presented in Fig. 1.

### ADRP identification

For ADRP identification, the standard SSM/PCA formulation was applied [21] to each of the 750 identification groups (25 replications of groups with 5 different sizes, each with 6 different image resolutions). A total of 750 versions of ADRP were obtained; for clarity, their names include a number of the identification subjects (20sub to 80sub), a consecutive number of the identification group with this particular size (1–25), and an image resolution in mm FWHM (6fwhm to 20fwhm). For example, ADRP-20sub-1-6fwhm for ADRP was identified from images of 20 AD patients and 20 CN, where the identification group was the first replication of this size, with an image resolution of 6 mm. A probabilistic gray matter mask was created by thresholding on 35% of the maximum voxel value. A specific disease-related pattern was determined as a PC or a linear combination of PCs, associated with maximum separation of AD patients’ and CN subjects’ scores, which were found with logistic regression that aims to find the lowest Akaike Information Criterion (AIC) value.

All 25 replications of the patterns identified from the identification groups with the same size and the same image resolution were averaged across spatially equivalent voxels. We obtained 30 average patterns, each characterized by a specific size of identification group and a specific resolution of identification images (e.g., ADRP-20sub-6fwhm). The average patterns were displayed and visually assessed.

### Diagnostic performance of ADRP

The area under the curve (AUC), calculated from the receiver operating characteristic (ROC) curve, was considered as a measure of the ADRP diagnostic performance. We used a voxel-based topographic profile rating (TPR) analysis [21] to calculate the expression of each of the 750 versions of ADRP in validation Cohort B at all six image resolutions. Consequently, for each of the 750 patterns, six ROC curves were determined, and six AUC values were calculated.

We compared AUC values across different identification group sizes, different resolutions of identification images, and different resolutions of validation images. For each group of 25 replications of ADRP characterized by the same identification group size and resolution of identification images (e.g., ADRP-20sub-1-6fwhm to ADRP-20sub-25-6fwhm), we calculated the average AUC and the average of the lowest five AUC values (i.e., the bottom 20% of all cases). This last value was considered an indicator of poor identification group selection, which may be a random outcome due to biological variability among the subjects. All the analyses were done with Matlab R2020a. SPM12 was used for creating a visual presentation of ADRP.

## Results

### ADRP identification

A total of 750 versions of ADRP were identified from the same number of identification groups. They were determined as different linear combinations of the first five PCs. Expression of all versions of ADRP in corresponding identification groups was abnormally elevated in AD patients compared to CN subjects (*p* < 0.001).

**Fig. 2:**
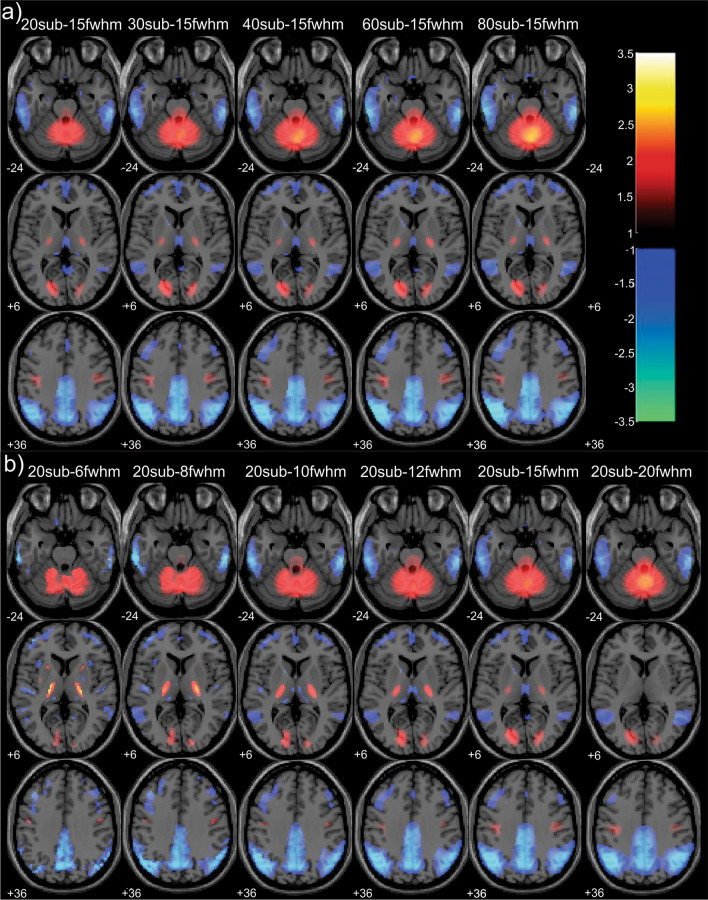
ADRP patterns. Examples of average ADRP patterns for **a** identification images with resolution 15 mm FWHM, for various sizes of identification groups: 20 AD/20 CN, 30 AD/30 CN, 40 AD/40 CN, 60 AD/60 CN and 80 AD/80 CN and **b** for identification group size 20 AD/20 CN, for image resolutions 6, 8, 10, 12, 15 and 20 mm. All displayed patterns are averaged over 25 replications of ADRP identified from the identification group of the same size and with the same image resolution. Red color represents metabolic hyperactivity, and blue color represents metabolic hypoactivity

Generally, the ADRP patterns were characterized by a relative decrease of metabolism in the precuneus, posterior cingulate cortex, anterior cingulate cortex, medial temporal lobe, and inferior parietal lobe and a relative increase of metabolism in the pons and cerebellum. Examples of average ADRPs, identified from images with resolution 15 mm FWHM for different sizes of identification groups (ADRP-20sub-15fwhm, ADRP-30sub-15fwhm, ADRP-40sub-15fwhm, ADRP-60sub-15fwhm and ADRP-80sub-15fwhm), are presented in Fig. 2a. Average ADRPs, identified from groups of 20 AD/20 CN for different image resolutions (ADRP-20sub-6fwhm, ADRP-20sub-8fwhm, ADRP-20sub-10fwhm, ADRP-20sub-12fwhm, ADRP-20sub-15fwhm, ADRP-20sub-20fwhm), are presented in Fig. 2b. Besides the regions presented in all ADRP versions, additional small hypermetabolic or hypometabolic regions are observed in individual versions of the pattern. For example, the hypermetabolic caudate nucleus is seen in versions of ADRP identified from images with a resolution of 6 mm FWHM, and the hypermetabolic thalamus is seen in ADRPs identified from images with a resolution of 6–15 mm (Fig. 2b). We can observe that patterns identified from images with better resolution show sharper details. Thirty average patterns for all sizes of identification groups and all image resolutions are in Appendix 3 (Fig. 6).

### Diagnostic performance of ADRP

**Fig. 3:**
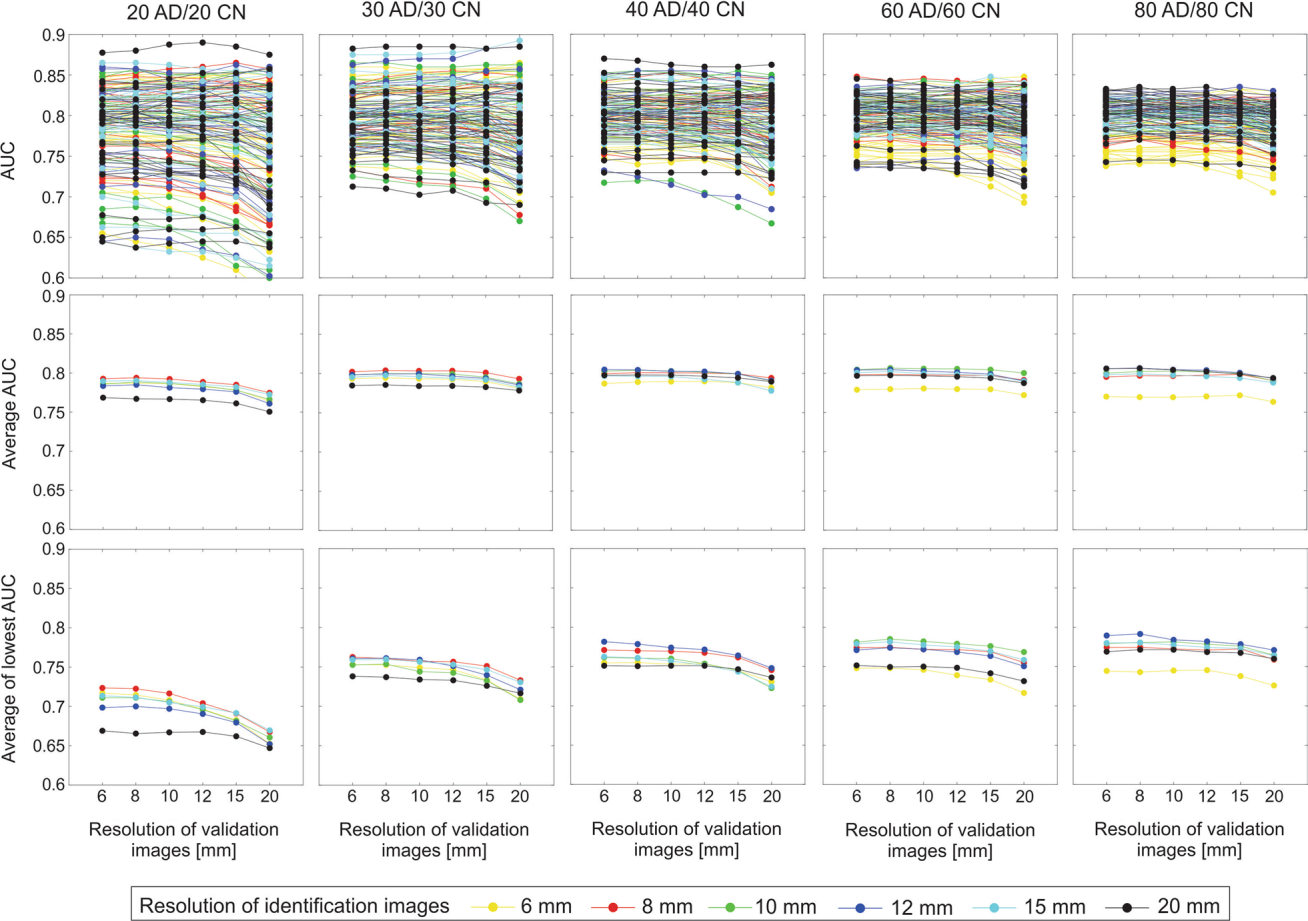
Dependence of AUC on the resolution of validation images for different sizes and resolutions of identification images*.* In the upper row, the plots show AUCs for all 25 replications of ADRP with the same size of identification group and for 6 resolutions of identification images (different colors). The average curves of 25 AUCs for each identification image resolution are in the middle row. In the bottom, row are the average curves of the lowest five AUCs for each resolution of identification images. Columns correspond to different identification group sizes. On the horizontal axes are different image resolutions of validation images. Different line colors represent different identification image resolutions

**Fig. 6:**
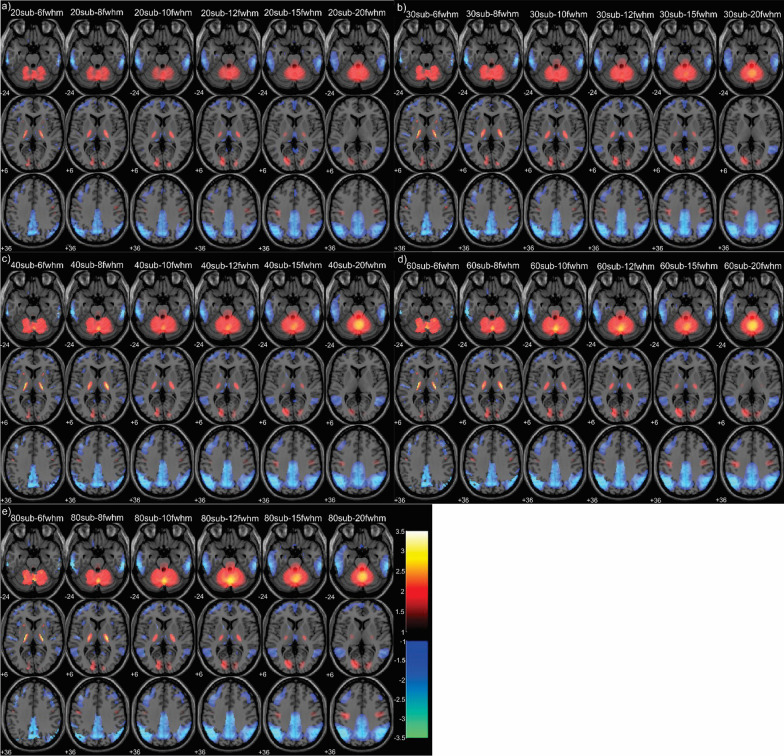
Display of average patterns identified from **a** 20 AD/20 CN, **b** 30 AD/30 CN, **c** 40 AD/40 CN, **d** 60 AD/60 CN, and **e** 80 AD/80 CN identification subjects

AUC values for different identification group sizes and resolutions of identification images, in dependence on the resolution of validation images, are presented in Fig. 3.

**Fig. 7:**
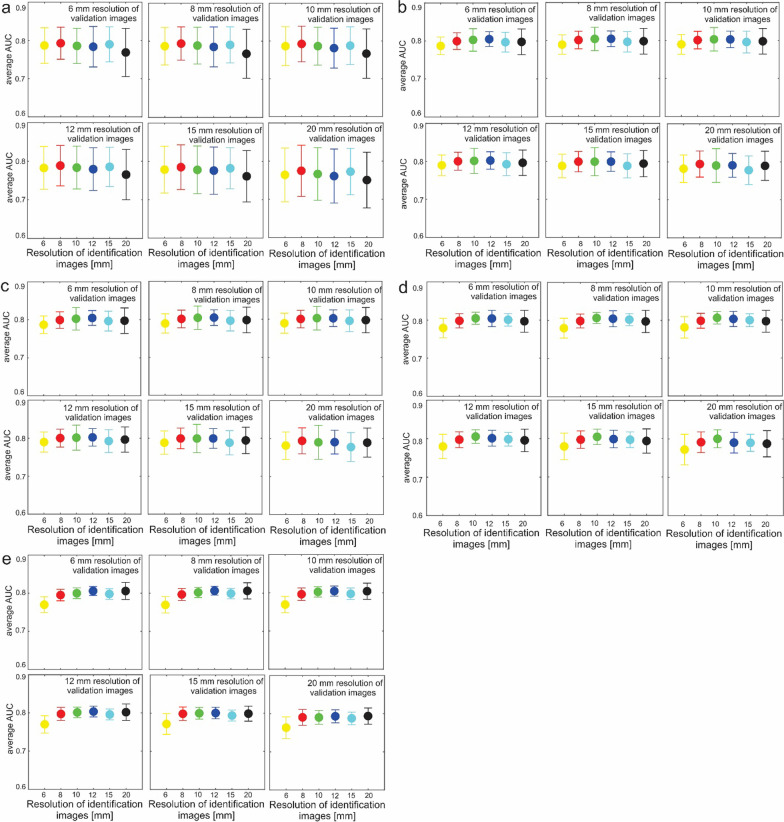
Average and standard deviation of AUC values across 25 different identification groups **a** 20 AD/20 CN, **b** 30 AD/30 CN, **c** 40 AD/40 CN, **d** 60 AD/60 CN and **e** 80 AD/80 CN. AUCs for patterns in six different image resolutions of identification and validation images are shown. Different colors represent different image resolutions in identification groups

AUCs for ADRPs with identification group size 20 AD/20 CN (Fig. 3, upper row) have a wide range of values (0.58–0.89), reflecting the biological variability, which is narrowing toward larger group sizes. For 30 AD/30 CN, the AUCs are 0.67–0.89; for 40 AD/40 CN, the AUCs are 0.67–0.87; for 60 AD/60 CN, the AUCs are 0.69–0.85; and for 80 AD/80 CN, the AUCs are 0.70–0.84. Standard deviations are 9% of AUC for 20 AD/20 CN, reducing to 2% and 3% for 60 AD/ 60 CN and 80 AD/80 CN, respectively. Mean values and standard deviations of AUCs for all 25 replications of ADRP with the same group size and resolution, describing the spread of the curves, are presented in Appendix 3 (Fig. 7).

In the plot of average AUC values (Fig. 3, middle row), an average behavior of ADRPs of a particular identification group size and image resolution can be observed. For identification group sizes 20 AD/20 CN and 30 AD/30 CN, image resolution 8 mm has the highest AUCs. For group sizes 40 AD/40 CN and 60 AD/60 CN, the 10 mm and 12 mm resolution patterns perform better (have higher AUC) than others. For identification group size 80 AD/80 CN, the patterns identified from images with resolutions 10 mm and 20 mm perform better than others. By comparing consecutive plots in the middle row of Fig. 2, we can see that increase in average AUC due to identification group size is only about 3% from size 20 AD/20 CN to 80 AD/80 CN.

The average of the lowest five AUC values (Fig. 3, bottom row) is the smallest for 20 AD/20 CN group size and increases substantially (for about 0.07) for 30 AD/30 CD group size for all image resolutions. An additional increase (about 0.02) is seen for 40 AD/40 CN, and then, the plateau is reached. The best performance is again at 8 mm for 20 AD/20 CN and 30 AD/30 CN and at 10 mm and 12 mm for larger identification group sizes. For small identification groups, the average of the lowest five AUC values is 10% lower compared to the average AUC value; for larger groups, the values are similar.

Regarding the changes in validation image resolution, we can see (Fig. 3, upper row) that the variations in AUCs are smaller than the variations in AUCs between the replications of the patterns with the same identification group sizes. The average AUC (Fig. 3, middle row) is very similar for validation images with resolutions from 6 to 15 mm but decreases (AUC decrease for about 0.01) for 20 mm in all group sizes and all resolutions of identification images. Similarly, the average of the 5 lowest AUCs (Fig. 3, bottom row) decreases toward 20 mm validation image resolution; the decrease is most pronounced in the 20 AD/20 CN group size (up to 0.03).

## Discussion

ADRP is a metabolic brain biomarker for AD and has potential for translation to clinical practice [8, 22, 23]. So far, various ADRP patterns have been identified and validated on different cohorts, from brain images acquired with different scanners, reconstructed with various algorithms, and smoothed with various filters. However, the effect of variation of these technical parameters on ADRP has not yet been thoroughly investigated. In this study, we systematically evaluated the impact of the size of the identification group and the impact of the image resolution of both identification and validation images on the ADRP’s diagnostic performance. The purpose of conducting such study was also to enable other researchers to estimate the ideal sample for their future SSM-based projects. The 2-[^18^F]FDG-PET images were randomly selected from the ADNI database. Since the ADNI database is an extensive multisite dataset, it is ideal for the derivation and validation of network biomarkers.

We identified 750 versions of ADRP and studied their AUC values for the discrimination of AD patients from healthy control subjects. All newly identified patterns had hypo- or hypermetabolic regions similar to those reported in previously published ADRPs [6, 7, 20, 24, 25]. Visually, the differences between patterns identified from identification groups that differed in sizes and resolutions were minor.

Nevertheless, we observed a wide spread of the AUC values among the 25 replications of ADRPs that were identified from the identification groups with the same sizes and image resolutions. These variations among AUCs were decreasing with the growing size of the identification group (at 20 AD/20 CN identification group size standard deviation of AUC was up to 9%, whereas at 60 AD/ 60 CN and 80 AD/80 CN, it dropped to 2% and 3%). This most likely implies that the biological variability among the subjects is less expressed in larger groups. By averaging AUC over the replications of the ADRPs identified from the same group size and image resolution of the identification images, we noticed that by increasing the size of the identification group, the best average AUC increased only slightly (from 20 AD/20 CN to 80 AD/80 CN subjects the AUC increased for about 3%). Generally, AUC values were the highest for medium resolution of the identification images (range from 8 to 15 mm) and were at these values also not sensitive to minor variations in image resolution. Larger groups favored worse identification image resolutions (for groups > 40 AD/40 CN the highest AUC is for FWHM > 10 mm, but for smaller groups, the highest AUC is at FWHM < 10 mm). Considering only the results of the average AUCs would mean that groups of 20 patients and 20 healthy controls can be sufficient for successful pattern identification. However, to reduce the effect of biological variability, using an identification group 30 AD/30 CN shall be beneficial. Our results also imply that smoothing out more details in the images when increasing the group size may produce a more robust pattern.

We further examined the average of the lowest five AUC values from the ADRPs with equal sizes of identification cohorts. For group sizes of 40 AD/40 CN and above, we could see that the behavior was very similar to the average AUC. Nevertheless, for smaller identification groups, especially for the 20 AD/20 CN one, the average of the lowest five AUC values is lower (about 10%) compared to the average AUC value. This suggests that the 20 AD/20 CN group size may be too small for a reliable pattern performance and that identification groups of size at least 30 AD/30 CN tend to have better performance. It also implies that due to biological variability or possible poor selection of subjects, a pattern identified from small cohorts could have compromised performance.

The performance of ADRP was not affected significantly by the resolution of validation images in prospective analyzes, implying that a properly identified ADRP can be used successfully with new images even if they have a different resolution than the identification images, as long as it is in the range between 6 and 15 mm.

In this study, we had a chance to observe an effect of biological variability demonstrated with group repetitions, and this could be compared to the performance of the previously published patterns. Perovnik et al. [8] report AUC values of 0.95 and 0.98 for ADRP identified from 20 AD/20 CN subjects and validated with two different sets of subjects. Iizuka et al. [25], with 50 AD/50 CN identification subjects, report AUC values between 0.80 and 1 for ten different samplings of identification and validation group. Mattis et al. [24] identified ADRP on 20 AD/20 CN ADNI data and obtained an AUC value of 0.86 for the identification and 0.87 for the validation cohort (personal communication). Two other authors report AUC values for the ADRP pattern but calculated only for identification subjects. Habeck et al. [4] compared the performance of five replications of ADRP, identified with different groups of 20 AD/20 CN subjects, and obtained AUC values between 0.87 and 0.97. Meles et al. [7] report an AUC value of 0.95 for ADRP identified on a small sample of 15 AD/18 CN subjects.

Our AUC values, calculated for validation subjects, are comparable to Iizuka et al. [25] and Mattis et al. [24] but lower than Perovnik et al. [8], whose ADRP has been identified in AD patients pathologically confirmed with a cerebrospinal fluid biomarker. Other authors report AUCs for the identification groups, which are expectedly higher than for validation groups. It could be seen that results that stem from ADNI data reach similar AUCs, probably due to the multisite nature of the data, and are different from the results obtained from single-center scans. Habeck et al. [4] and Iizuka et al. [25] also confirm the spread of AUC values for group repetitions and imply that the selection of the subjects may have a significant impact on the AUC value. The reason for somehow lower AUC values in our study and their wider spread, especially in smaller identification groups, is possibly caused by the random selection of subjects from the ADNI database. In the selection process, we considered patients’ diagnoses but not their detailed clinical data (i.e., disease duration, cognitive status), which could improve subject selection. It should also be noted that subjects in our study were scanned on four different scanners with considerably different configurations (whole-body PET scanner vs. dedicated brain PET scanner) and different scanner generations. Due to these differences, images were likely more heterogeneous regarding noise level, and these differences could not be fully corrected with scanner-specific image smoothing. Additionally, it should be emphasized that our reported AUC values stem from the validation images, while others generally reported AUC values that stem from the identification image sets, which may cause a possible overfitting bias.

Our findings about the appropriate resolution of the ADRP identification images can be roughly compared to the image resolutions chosen by the authors in previous studies. Smoothing with 10 mm [6, 7, 20, 24] or 12 mm [4, 5] FWHM Gaussian kernel is reported, leading to the image resolution of about 11.4 mm or 13.2 mm FWMH for Siemens Biograph mCT and worse for other scanners. This is compliant with the highest AUCs for patterns identified from images with resolutions between 8 and 15 mm FWHM in our study.

Nevertheless, we found that the effect of image resolution on the pattern’s diagnostic performance is rather small, which is in accordance with previous research exploring the influence of other technical parameters of the 2-[^18^F]FDG-PET images on other disease-specific metabolic patterns. In Tomše et al. [15], we estimated the effect of 2-[^18^F]FDG-PET reconstruction algorithms on the expression of the Parkinson’s disease-related pattern (PDRP). The reported AUC values for differentiation between patients and healthy controls are stable regardless of the reconstruction algorithm; around 0.95. Even so, it was stressed that for the prospective determination of pattern expression in a new subject, scaling with a group of CN subjects’ images scanned and preprocessed with the same protocol is needed. Similarly, stable AUC values were achieved for PDRP also by Peng et al. [18], who studied the effects of PET scanners and spatial normalization with different softwares and reported AUC values between 0.96 and 0.97 for different SPM versions. Wu et al. [16] studied PDRP scores in two populations scanned with different scanners and reconstruction algorithms and obtained AUC values between 0.98 and 0.99. Moeller et al. [17] also confirmed a high reproducibility of the PDRPs across four independent pattern identification populations each scanned with a different PET scanner.

Our study has some limitations. Firstly, random machine selection of patients’ images from the publicly available ADNI database might differ from the recruitment of patients to the prospective studies with the original purpose of disease-specific pattern identification. From the database, the patients were selected according to their clinical diagnosis, which may be wrong in around one-third of patients [26]. Additionally, our data on the scanner’s characteristics were limited to the type of scanner and the reported effective resolution, which we then used to calculate the filter’s FWHM needed to reach the final image resolution. Other factors can play a role in the quality of the images, such as image count rates, depending upon injected radioactivity, body mass, blood glucose level, medications, possible sleeping, room lighting, ambient sound levels, and staff interactions with the subjects. Image resolution can also be affected by motion artifacts.

Further assessments of ADRP’s diagnostic performance should check whether AUCs from a single site testing set are systematically greater than analogous values from the multisite data, e.g., ADNI, and whether AUCs would increase over time as AD progresses.

## Conclusions

In this study, we systematically addressed the effects of the number of 2-[^18^F]FDG-PET images in the identification group and the effect of the image resolution in the identification and validation groups on the diagnostic performance of ADRP.

We showed that an average AUC of the ROC curve for the differentiation between AD patients and healthy controls only marginally increases in larger (more than 20 AD/20 CN) identification subject groups. However, in case of a poorer selection of patients, the AUC increases with an increasing number of participants. Therefore, to reduce the effect of biological variability, an identification group of 30 AD/30 CN or a larger size shall be beneficial. We believe that this result shall be applied to the identification of other metabolic patterns using SSM/PCA analysis.

Based on our findings, the identification image resolution should stay within a range of 8–15 mm. SSM/PCA-based network patterns can be applied to images acquired with different scanners yielding different image resolutions, if it is within the range of 6–15 mm.

## Figures and Tables

**Fig. 4:**
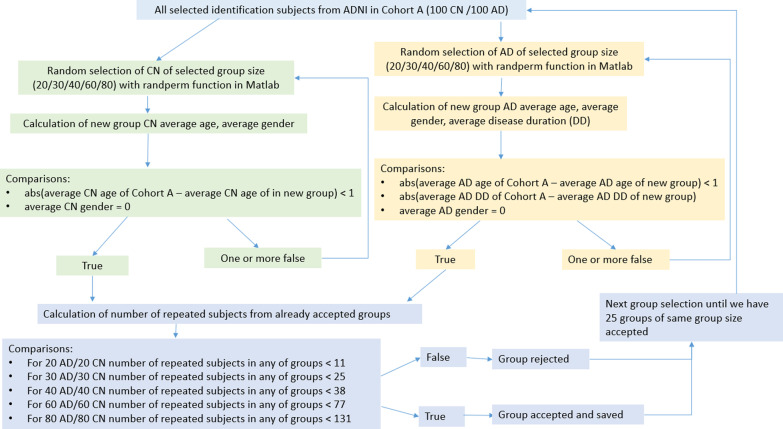
Algorithm for assigning subjects to different identification groups

**Fig. 5:**
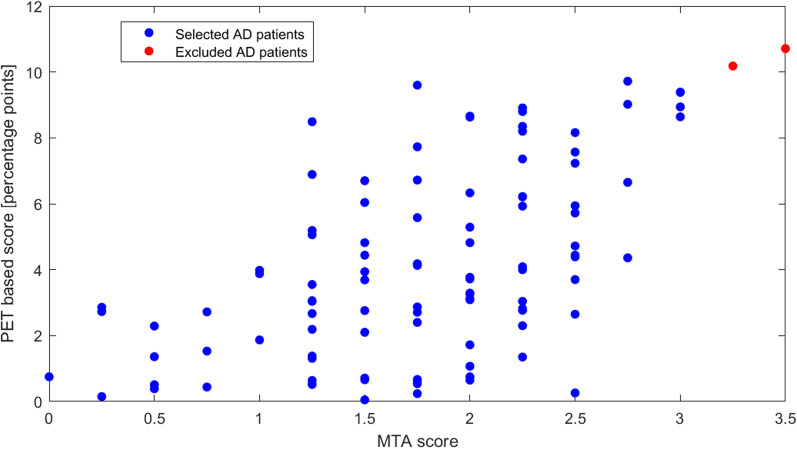
Correlation plot between the MTA score and our PET-based score

## Data Availability

The datasets used for the study are available on the ADNI database. Code from the study is available from the corresponding author on reasonable request.

## Appendix 1: groups selection

A total of 125 groups from Cohort A, comprised of 100 AD and 100 CN with random selection, were produced. The first 25 groups consisted of 20 AD patients and 20 normal controls (CN), which were balanced in gender, and had similar average age and disease duration. The second 25 groups consisted of 30 AD/30 CN subjects, the third 25 groups consisted of 40 AD/40 CN subjects, the fourth 25 groups consisted of 60 AD/60 CN subjects, and the fifth 25 groups consisted of 80 AD/80 CN subjects. Algorithm for assigning subjects to different identification groups randomly assigned patients to individual subgroups until average age and disease duration differed less than 1 year and 0.5 from average age and disease duration of the whole Cohort A. Algorithm also checked the number of repeated subjects in different subgroups and if the number of repeated subjects was greater than some threshold (for different groups sizes different) the new subgroup was rejected and another selection was done. Algorithm is presented in Fig. 4. Demographic data for each group are presented in Tables 3, 4, 5, 6, 7.

**Table 3:** Demographic data of 20AD/20 CN identification groups

	Group 1		Group 2		Group 3		Group 4		Group 5	
	CN	AD	CN	AD	CN	AD	CN	AD	CN	AD
Age [years]	76.3 ± 5.9	76.5 ± 6.5	76.8 ± 5.6	76.6 ± 5.9	77.1 ± 6.2	76.7 ± 6.9	76.6 ± 6.3	76.7 ± 7.3	76.2 ± 6.2	77.0 ± 8.8
DD [years]	–	5.0 ± 3.1	–	5.1 ± 3.0	–	5.1 ± 2.8	–	5.1 ± 2.8	–	5.2 ± 2.5
MMSE	28.6 ± 1.5	23.4 ± 3.0	28.6 ± 1.3	23.9 ± 2.2	28.8 ± 1.2	22.7 ± 2.9	29.1 ± 1.4	23.3 ± 3.8	28.6 ± 1.8	24.5 ± 2.5

	Group 6		Group 7		Group 8		Group 9		Group 10	
	CN	AD	CN	AD	CN	AD	CN	AD	CN	AD
Age [years]	76.8 ± 5.8	76.9 ± 5.1	76.9 ± 6.9	76.2 ± 7.9	77.0 ± 4.0	77.0 ± 8.7	76.3 ± 7.0	76.9 ± 6.3	77.0 ± 7.3	76.9 ± 5.1
DD [years]	–	5.1 ± 3.0	–	5.3 ± 3.1	–	5.3 ± 2.4	–	5.2 ± 2.9	–	5.0 ± 3.0
MMSE	28.5 ± 2.4	24.9 ± 2.1	28.8 ± 1.0	24.0 ± 2.0	28.7 ± 1.3	22.8 ± 3.7	28.8 ± 1.2	22.2 ± 2.7	29.0 ± 1.4	23.8 ± 2.8

	Group 11		Group 12		Group 13		Group 14		Group 15	
	CN	AD	CN	AD	CN	AD	CN	AD	CN	AD
Age [years]	77.0 ± 7.2	76.8 ± 8.4	76.4 ± 5.2	76.4 ± 6.2	76.7 ± 6.3	76.3 ± 7.1	76.4 ± 7.1	76.9 ± 6.3	77.0 ± 5.1	77.0 ± 6.0
DD [years]	–	5.0 ± 2.8	–	4.9 ± 2.7	–	5.0 ± 2.7	–	5.2 ± 2.4	–	4.9 ± 2.4
MMSE	29.4 ± 0.9	23.1 ± 3.3	29.0 ± 1.5	23.6 ± 2.7	28.5 ± 1.3	22.3 ± 3.5	28.5 ± 2.4	22.3 ± 3.3	28.7 ± 1.7	23.8 ± 2.7

	Group 16		Group 17		Group 18		Group 19		Group 20	
	CN	AD	CN	AD	CN	AD	CN	AD	CN	AD
Age [years]	77.1 ± 5.8	76.9 ± 7.2	76.6 ± 4.9	76.9 ± 5.7	76.7 ± 6.8	77.0 ± 6.1	76.3 ± 7.1	77.0 ± 5.5	76.4 ± 6.1	75.5 ± 5.6
DD [years]	–	5.2 ± 2.9	–	4.9 ± 2.6	–	4.9 ± 3.2	–	4.9 ± 3.8	–	5.0 ± 2.5
MMSE	28.3 ± 2.3	22.8 ± 1.8	28.4 ± 1.8	24.4 ± 3.5	28.9 ± 1.4	23.7 ± 2.5	29.1 ± 1.5	22.2 ± 3.0	29.3 ± 1.1	23.1 ± 2.2

	Group 21		Group 22		Group 23		Group 24		Group 25	
	CN	AD	CN	AD	CN	AD	CN	AD	CN	AD
Age [years]	76.9 ± 7.4	77.1 ± 6.2	76.5 ± 6.2	77.0 ± 7.7	76.8 ± 6.4	76.7 ± 7.6	76.7 ± 5.5	76.9 ± 6.1	76.5 ± 6.3	76.3 ± 8.1
DD [years]	–	5.0 ± 3.1	–	5.3 ± 4.5	–	5.1 ± 2.6	–	5.3 ± 3.1	–	5.1 ± 2.7
MMSE	28.3 ± 1.3	23.6 ± 2.8	28.5 ± 2.5	24.3 ± 3.6	28.3 ± 1.9	23.0 ± 2.7	28.9 ± 1.4	23.7 ± 2.5	28.8 ± 1.3	23.6 ± 2.8

**Table 4:** Demographic data of 30 AD/30 CN identification groups

	Group 26		Group 27		Group 28		Group 29		Group 30	
	CN	AD	CN	AD	CN	AD	CN	AD	CN	AD
Age [years]	77.0 ± 6.1	76.2 ± 6.5	77.0 ± 6.3	75.8 ± 6.5	75.8 ± 6.1	77.4 ± 7.4	76.2 ± 5.9	77.5 ± 7.2	76.3 ± 5.4	77.3 ± 4.9
DD [years]		4.7 ± 2.7		4.9 ± 2.6		5.2 ± 2.5		5.3 ± 3.8		4.7 ± 3.0
MMSE	28.9 ± 1.2	23.8 ± 3.7	28.6 ± 2.1	23.8 ± 2.6	29.0 ± 1.1	22.2 ± 2.9	28.6 ± 1.3	22.7 ± 2.7	28.7 ± 1.5	23.1 ± 2.8

	Group 31		Group 32		Group 33		Group 34		Group 35	
	CN	AD	CN	AD	CN	AD	CN	AD	CN	AD
Age [years]	76.3 ± 5.0	76.1 ± 6.9	76.2 ± 7.1	77.0 ± 4.8	76.3 ± 6.6	76.1 ± 7.2	76.0 ± 5.7	77.4 ± 7.6	76.9 ± 4.9	75.7 ± 6.5
DD [years]		4.6 ± 2.6		4.7 ± 3.3		4.7 ± 2.5		5.3 ± 3.1		4.8 ± 3.0
MMSE	28.7 ± 1.4	23.6 ± 3.5	28.8 ± 1.3	23.3 ± 3.0	28.5 ± 2.1	23.3 ± 2.6	28.7 ± 1.3	23.2 ± 3.5	28.8 ± 1.4	23.1 ± 3.4

	Group 36		Group 37		Group 38		Group 39		Group 40	
	CN	AD	CN	AD	CN	AD	CN	AD	CN	AD
Age [years]	76.5 ± 6.2	76.0 ± 6.6	76.6 ± 6.3	76.6 ± 7.3	77.6 ± 6.2	77.5 ± 7.1	77.5 ± 6.4	75.7 ± 6.5	76.3 ± 5.8	76.9 ± 6.7
DD [years]		4.9 ± 3.0		4.8 ± 3.1		4.7 ± 2.6		4.9 ± 2.9		5.6 ± 3.7
MMSE	28.0 ± 2.1	23.7 ± 3.0	28.5 ± 1.5	22.9 ± 2.6	28.6 ± 2.0	23.6 ± 2.8	28.5 ± 1.5	23.5 ± 3.2	28.8 ± 1.6	22.6 ± 3.7

	Group 41		Group 42		Group 43		Group 44		Group 45	
	CN	AD	CN	AD	CN	AD	CN	AD	CN	AD
Age [years]	76.0 ± 6.7	75.9 ± 7.3	76.6 ± 5.1	76.7 ± 6.4	76.1 ± 6.5	76.4 ± 6.2	76.5 ± 6.9	77.4 ± 8.2	77.1 ± 6.1	77.4 ± 8.3
DD [years]		5.4 ± 3.0		5.1 ± 2.5		4.7 ± 3.4		5.1 ± 3.0		5.5 ± 3.1
MMSE	28.9 ± 1.3	23.3 ± 3.0	28.6 ± 2.0	22.7 ± 3.0	28.7 ± 1.3	23.1 ± 2.9	28.3 ± 2.0	22.8 ± 3.1	27.8 ± 2.3	22.9 ± 3.9

	Group 46		Group 47		Group 48		Group 49		Group 50	
	CN	AD	CN	AD	CN	AD	CN	AD	CN	AD
Age [years]	76.7 ± 6.0	75.8 ± 5.6	76.1 ± 6.9	76.1 ± 5.5	76.3 ± 6.0	76.9 ± 5.9	76.8 ± 6.4	76.1 ± 4.7	76.6 ± 6.3	75.9 ± 7.8
DD [years]		5.1 ± 3.7		5.5 ± 3.1		5.1 ± 2.8		5.1 ± 3.3		5.0 ± 3.6
MMSE	28.6 ± 1.5	23.4 ± 2.6	28.4 ± 2.2	23.7 ± 2.9	28.7 ± 1.3	23.2 ± 2.8	29.0 ± 1.2	23.1 ± 2.4	28.6 ± 1.7	23.1 ± 3.0

**Table 5:** Demographic data of 40 AD/40 CN identification groups

	Group 51		Group 52		Group 53		Group 54		Group 55	
	CN	AD	CN	AD	CN	AD	CN	AD	CN	AD
Age [years]	76.4 ± 6.0	76.7 ± 6.8	76.6 ± 5.8	77.0 ± 5.9	76.5 ± 5.9	77.2 ± 7.0	76.7 ± 5.8	76.8 ± 6.0	76.1 ± 6.1	76.6 ± 6.0
DD [years]		5.4 ± 2.8		4.8 ± 3.0		5.5 ± 3.2		4.9 ± 2.7		5.0 ± 3.1
MMSE	28.7 ± 2.0	22.6 ± 2.7	28.9 ± 1.4	23.9 ± 2.3	28.67 ± 1.5	23.0 ± 3.6	28.7 ± 2.1	23.0 ± 2.9	28.6 ± 1.4	23.3 ± 2.6

	Group 56		Group 57		Group 58		Group 59		Group 60	
	CN	AD	CN	AD	CN	AD	CN	AD	CN	AD
Age [years]	77.1 ± 5.5	76.1 ± 7.2	76.3 ± 6.3	76.3 ± 7.5	76.5 ± 5.8	75.9 ± 7.1	77.0 ± 6.3	76.4 ± 6.2	76.8 ± 6.3	77.4 ± 7.2
DD [years]		5.1 ± 3.3		5.4 ± 3.0		5.2 ± 2.9		4.7 ± 2.6		4.9 ± 2.9
MMSE	28.8 ± 1.2	23.6 ± 2.7	28.4 ± 1.6	23.4 ± 2.5	28.6 ± 1.9	22.9 ± 3.0	28.6 ± 1.4	23.4 ± 2.9	28.3 ± 2.0	23.2 ± 3.1

	Group 61		Group 62		Group 63		Group 64		Group 65	
	CN	AD	CN	AD	CN	AD	CN	AD	CN	AD
Age [years]	76.6 ± 6.0	75.9 ± 6.6	77.2 ± 5.9	76.7 ± 7.0	75.7 ± 5.7	76.5 ± 7.0	75.9 ± 6.3	76.3 ± 6.6	77.3 ± 5.9	76.8 ± 7.2
DD [years]		5.0 ± 2.8		5.0 ± 2.9		4.6 ± 2.6		5.2 ± 3.4		4.6 ± 2.5
MMSE	28.6 ± 1.4	23.0 ± 2.6	28.8 ± 1.8	22.9 ± 3.7	28.9 ± 1.3	23.7 ± 2.9	28.7 ± 2.0	23.2 ± 3.0	28.6 ± 2.0	23.1 ± 3.3

	Group 66		Group 67		Group 68		Group 69		Group 70	
	CN	AD	CN	AD	CN	AD	CN	AD	CN	AD
Age [years]	76.8 ± 5.9	77.4 ± 6.9	76.7 ± 5.8	76.8 ± 7.7	77.0 ± 5.9	76.7 ± 7.1	77.4 ± 6.1	76.3 ± 5.5	76.3 ± 5.6	76.6 ± 6.8
DD [years]		4.7 ± 2.6		5.5 ± 3.7		5.0 ± 3.0		4.9 ± 2.9		5.2 ± 2.7
MMSE	28.7 ± 1.4	22.2 ± 3.2	28.5 ± 2.0	22.8 ± 3.5	28.5 ± 1.5	23.6 ± 2.5	28.8 ± 1.3	23.8 ± 3.0	28.5 ± 2.0	22.8 ± 3.8

	Group 71		Group 72		Group 73		Group 74		Group 75	
	CN	AD	CN	AD	CN	AD	CN	AD	CN	AD
Age [years]	75.9 ± 6.0	76.5 ± 7.2	77.0 ± 5.8	76.0 ± 6.7	76.1 ± 6.2	77.0 ± 7.5	76.0 ± 5.5	75.6 ± 7.4	76.8 ± 6.4	77.3 ± 6.6
DD [years]		5.2 ± 3.3		5.1 ± 3.1		5.6 ± 3.2		4.6 ± 2.5		4.9 ± 2.6
MMSE	28.4 ± 2.0	23.6 ± 3.0	28.6 ± 1.6	23.0 ± 2.9	28.7 ± 1.5	23.2 ± 3.4	28.8 ± 1.4	23.4 ± 3.5	29.0 ± 1.8	23.1 ± 3.0

**Table 6:** Demographic data of 60 AD/60 CN identification groups

	Group 76		Group 77		Group 78		Group 79		Group 80	
	CN	AD	CN	AD	CN	AD	CN	AD	CN	AD
Age [years]	76.5 ± 6.5	75.9 ± 7.0	76.9 ± 5.6	76.2 ± 6.4	77.0 ± 6.0	76.0 ± 7.1	76.1 ± 6.3	76.4 ± 5.6	77.0 ± 5.6	76.6 ± 7.1
DD [years]		5.2 ± 3.3		5.3 ± 3.1		5.0 ± 3.1		5.0 ± 3.1		5.1 ± 3.3
MMSE	28.7 ± 1.3	23.6 ± 2.5	28.6 ± 1.4	23.2 ± 2.8	28.9 ± 1.4	23.2 ± 3.1	28.9 ± 1.3	23.6 ± 2.7	29.0 ± 1.3	23.0 ± 3.0

	Group 81		Group 82		Group 83		Group 84		Group 85	
	CN	AD	CN	AD	CN	AD	CN	AD	CN	AD
Age [years]	76.1 ± 6.2	77.7 ± 6.8	75.8 ± 6.2	76.3 ± 6.6	76.3 ± 6.7	76.6 ± 7.1	77.0 ± 5.9	76.1 ± 6.6	77.2 ± 5.5	76.4 ± 7.3
DD [years]		5.4 ± 3.4		5.1 ± 3.4		5.5 ± 3.1		4.7 ± 2.5		5.2 ± 3.3
MMSE	28.5 ± 1.8	23.2 ± 2.8	28.7 ± 1.7	23.4 ± 2.4	28.5 ± 1.9	23.5 ± 2.7	28.8 ± 1.3	23.2 ± 2.7	28.8 ± 1.4	23.3 ± 3.4

	Group 86		Group 87		Group 88		Group 89		Group 90	
	CN	AD	CN	AD	CN	AD	CN	AD	CN	AD
Age [years]	76.2 ± 6.2	76.9 ± 7.2	76.6 ± 5.3	76.0 ± 6.8	77.2 ± 5.6	76.0 ± 6.3	76.6 ± 5.7	76.9 ± 6.6	76.0 ± 6.2	75.9 ± 6.8
DD [years]		5.2 ± 2.9		5.3 ± 3.2		4.6 ± 2.6		4.7 ± 2.6		5.3 ± 3.2
MMSE	28.8 ± 1.4	23.0 ± 3.3	28.7 ± 1.8	23.7 ± 2.8	28.7 ± 1.8	23.4 ± 3.2	28.7 ± 1.8	23.1 ± 3.3	28.6 ± 1.8	23.3 ± 3.3

	Group 91		Group 92		Group 93		Group 94		Group 95	
	CN	AD	CN	AD	CN	AD	CN	AD	CN	AD
Age [years]	76.0 ± 6.1	76.4 ± 6.6	77.0 ± 5.5	76.3 ± 6.8	76.3 ± 6.3	76.7 ± 7.0	76.3 ± 6.1	75.7 ± 6.6	76.5 ± 5.9	77.1 ± 6.9
DD [years]		5.6 ± 3.3		5.3 ± 3.2		5.2 ± 2.9		4.8 ± 3.1		5.0 ± 2.9
MMSE	28.6 ± 1.8	23.2 ± 3.1	28.5 ± 1.8	23.1 ± 2.8	28.4 ± 1.8	23.0 ± 3.0	28.8 ± 1.3	23.3 ± 3.1	28.6 ± 1.8	23.5 ± 3.1

	Group 96		Group 97		Group 98		Group 99		Group 100	
	CN	AD	CN	AD	CN	AD	CN	AD	CN	AD
Age [years]	76.7 ± 6.4	76.5 ± 7.2	76.8 ± 6.2	75.7 ± 6.9	76.4 ± 5.8	76.7 ± 7.0	76.8 ± 5.9	77.1 ± 6.5	76.7 ± 6.0	77.0 ± 6.7
DD [years]		4.9 ± 3.0		5.1 ± 3.2		5.0 ± 3.1		5.3 ± 3.2		5.2 ± 2.8
MMSE	28.6 ± 1.8	23.2 ± 2.9	28.8 ± 1.7	23.5 ± 2.5	29.0 ± 1.2	23.3 ± 2.9	28.6 ± 1.8	23.1 ± 3.1	28.6 ± 1.9	23.1 ± 3.2

**Table 7:** Demographic data of 80 AD/80 CN identification groups

	Group 101		Group 102		Group 103		Group 104		Group 105	
	CN	AD	CN	AD	CN	AD	CN	AD	CN	AD
Age [years]	76.8 ± 6.3	76.8 ± 6.9	76.2 ± 6.3	76.5 ± 6.5	76.3 ± 6.2	76.6 ± 6.9	75.8 ± 5.9	76.0 ± 6.2	76.8 ± 5.7	76.7 ± 7.1
DD [years]		5.2 ± 3.2		5.0 ± 3.1		5.2 ± 3.1		5.0 ± 3.1		5.3 ± 3.2
MMSE	28.5 ± 1.7	23.3 ± 2.8	28.8 ± 1.6	23.1 ± 3.0	28.7 ± 1.7	23.1 ± 3.1	28.7 ± 1.7	23.1 ± 2.9	28.7 ± 1.5	23.1 ± 3.1

	Group 106		Group 107		Group 108		Group 109		Group 110	
	CN	AD	CN	AD	CN	AD	CN	AD	CN	AD
Age [years]	75.6 ± 5.7	76.3 ± 6.6	76.3 ± 6.1	76.4 ± 6.5	76.8 ± 6.3	76.6 ± 6.8	76.5 ± 6.1	77.1 ± 6.6	76.8 ± 6.1	77.1 ± 6.7
DD [years]		5.0 ± 3.2		5.0 ± 2.7		5.0 ± 2.8		4.8 ± 2.7		5.2 ± 3.2
MMSE	28.6 ± 1.7	23.3 ± 3.0	28.6 ± 1.8	23.3 ± 2.6	28.7 ± 1.7	23.2 ± 3.0	28.7 ± 1.7	23.0 ± 3.0	28.6 ± 1.7	23.4 ± 3.0

	Group 111		Group 112		Group 113		Group 114		Group 115	
	CN	AD	CN	AD	CN	AD	CN	AD	CN	AD
Age [years]	77.0 ± 6.0	76.6 ± 6.8	76.3 ± 6.0	76.7 ± 6.5	76.5 ± 5.8	77.3 ± 6.4	76.9 ± 5.9	76.4 ± 6.8	76.6 ± 6.1	76.2 ± 7.0
DD [years]		5.0 ± 3.1		5.3 ± 3.1		4.8 ± 3.1		5.2 ± 3.0		5.1 ± 3.1
MMSE	28.6 ± 1.7	23.4 ± 2.9	28.7 ± 1.6	23.2 ± 3.1	28.7 ± 1.8	23.0 ± 2.9	28.6 ± 1.7	23.3 ± 3.0	28.7 ± 1.5	23.3 ± 3.1

	Group 116		Group 117		Group 118		Group 119		Group 120	
	CN	AD	CN	AD	CN	AD	CN	AD	CN	AD
Age [years]	76.4 ± 6.3	76.1 ± 6.8	76.5 ± 6.3	76.4 ± 6.7	77.2 ± 6.1	76.0 ± 6.8	76.0 ± 6.0	76.2 ± 7.0	76.5 ± 5.8	76.3 ± 7.0
DD [years]		5.2 ± 3.1		4.9 ± 3.1		4.5 ± 2.4		5.0 ± 2.6		5.0 ± 3.0
MMSE	28.6 ± 1.7	23.1 ± 3.0	28.7 ± 1.6	23.4 ± 2.9	28.7 ± 1.6	23.2 ± 2.9	28.7 ± 1.7	23.3 ± 3.1	28.7 ± 1.5	23.2 ± 3.0

	Group 121		Group 122		Group 123		Group 124		Group 125	
	CN	AD	CN	AD	CN	AD	CN	AD	CN	AD
Age [years]	77.0 ± 6.3	76.2 ± 6.9	77.1 ± 6.2	76.6 ± 6.7	76.7 ± 5.9	76.1 ± 6.4	76.7 ± 6.2	76.8 ± 6.8	76.6 ± 5.9	76.9 ± 6.7
DD [years]		5.2 ± 3.2		5.3 ± 3.1		5.0 ± 3.1		4.9 ± 2.8		4.9 ± 3.0
MMSE	28.6 ± 1.7	23.1 ± 2.8	28.8 ± 1.4	23.1 ± 3.1	28.6 ± 1.8	23.6 ± 3.0	28.8 ± 1.4	23.3 ± 3.2	28.8 ± 1.3	23.3 ± 3.2

It should be noted that the threshold for the number of matching subjects, which is higher for larger subgroups, does not constitute an inherent source of selection bias. We empirically determined the (approximate) lowest threshold for the maximal number of repeated subjects. We started a subgroup selection with a certain small number of repeated subjects (explained later) and if the solution could not be found in a reasonable time (a few hours), we relaxed a criterion for the number of repeated subjects until we got a solution. Generally, if we have n subjects and we try to get a combination with k subjects, we have C(n, k) possible combinations (binomial coefficient). When taking all the combinations, each particular combination has up to (k-1) common subjects with any other combination (at least one subject must be different). We can select a subset of all possible combinations, where more than one subject is different in any pair of selected combinations. But there are limitations to that; for example, if k > n/2, we can’t find two combinations with zero common subjects. It can be shown that two distinct combinations with k subjects, drawn from a set of n subjects have at least 2 k-n common subjects. This gives us the lower limit of 20 common subjects when 60 subjects is drawn out of 100 subjects and 60 common subjects when 80 subjects is drawn out of 100 subjects. For 20, 30 and 40 subjects drawn out of 100 subjects, we can have some combinations with no common subjects. Those were initial limits for the number of repeated subjects in the groups selection algorithm. However, the number of repeated subjects may be higher when more than two combinations are drawn. In addition to that, our selected subgroups were not just random subgroups, but we also required that all subgroups matched in average age and gender. Therefore, the final number of repeated subjects were higher than the 2 k-n.

## Appendix 2: atrophy screening

PET-based algorithm for atrophy screening assesses relative size of the cerebrospinal fluid compartment for each prospective AD patient and compare it to the assessment for the average 2-[^18^F]FDG-PET image of 20 healthy controls. Relative size of the cerebrospinal fluid was assessed by the following procedure:

- the brain is segmented with the help of an atlas,
- within the brain segment from the atlas, those voxels that are below 30% of the maximum 2-[^18^F]FDG-PET value are considered to represent cerebrospinal fluid,
- the proportion of brain segment that consists of the cerebrospinal fluid is calculated to get the relative size of the cerebrospinal fluid (measured in percents).

If the relative size of the cerebrospinal compartment for prospective AD patient was higher than the relative size of the cerebrospinal compartment for average normal control for more than 10 percentage points, the prospective AD patient was excluded due to excessive atrophy. We arbitrarily defined a threshold of 10 percentage points as we empirically observed that patients with severe atrophy had the relative size of the cerebrospinal compartment that exceed by more than 10 percentage points the relative size of normal controls.

In order to verify our PET-based atrophy screening algorithm, we evaluated MTA scores for those patients, where the MR image was available. Out of 120 AD patients, 101 had a MR image available. Their MTA scores range between 0 and 3. For 41 AD patients out of 101, their MTA score was abnormal, but still none of them had MTA score above 3. We correlated the MTA score with our PET-based percentage score and found a reasonable correlation of the two atrophy measures (please see the correlation plot in Fig. 5). When we selected patients with our method, only three patients were excluded due to excessive (above 10%) atrophy. Two of them had MR images, and their MTA scores were indeed higher than the MTA scores of the studied patients, i.e., 3.25 and 3.5 (red spots in Fig. 5).

We can observe in Fig. 5 that the worst agreement between the methods is in the middle range of MTA values. Patients with low MTA (≤ 1) have consistently low PET score (< 4%), and patients with higher MTA (> 3) have PET score consistently above 8%.

## Appendix 3: patterns identified from different sizes of identification subject groups and different image resolutions

All average patterns displayed for 20 AD/20 CN, 30 AD/30 CN, 40 AD/40 CN, 60 AD/60 CN, and 80 AD/80 CN identification groups are shown in Figs. 6, 7 presents the average AUC value with standard deviation for all sizes of identification cohorts. It could be seen that with a growing number of identification cohorts, standard deviations are getting smaller.

## Abbreviations

AD: Alzheimer’s disease
ADRP: Alzheimer’s disease-related pattern
CN: Cognitively normal
SSM/PCA: Scaled subprofile model/principal component analysis
2-[^18^F]FDG-PET: 2-[^18^F]fluoro-2-deoxy-d-glucose positron emission tomography
PC: Principal component
FWHM: Full width at half maximum
AUC: Area under the curve
ROC: Receiver operating characteristic
